# Psychological needs and support among patients and families undergoing food oral immunotherapy

**DOI:** 10.1002/clt2.12078

**Published:** 2022-02-03

**Authors:** Laura Polloni, Antonella Muraro, Roberta Bonaguro, Alice Toniolo, Anna Ballin, Alberto Guarnaccia, Francesca Lazzarotto

**Affiliations:** ^1^ Department of Women's and Children's Health Food Allergy Referral Centre, Veneto Region Padua University Hospital Padua Italy; ^2^ Psychology Unit Padua University Hospital Padua Italy; ^3^ Faculty of Medicine and Surgery Vita‐Salute San Raffaele University Milan Italy

**Keywords:** adolescents, anaphylaxis, anxiety, children, compliance, coping, counselling, diet, food allergy, immunotherapy, parents, psychotherapy, anaphylaxie, angst, compliance, coping, counselling, diät, eltern, immuntherapie, jugendliche, kinder, nahrungsmittelallergie, psychotherapie

## Abstract

**Background:**

Oral immunotherapy (OIT) is a promising treatment for food allergy (FA) however it is a challenging process for patients and parents. Induction can generate stress and anxiety. This may in turn affect their motivation and ability to cope with OIT challenges.

**Objective:**

This study aimed to investigate psychological needs and support to patients/parents undergoing food OIT assessing participants' main characteristics, reasons for seeking psychological support, OIT phase and related psychological difficulties, type and timing of treatments and patients' perception of the effectiveness of the intervention.

**Methods:**

This is an observational, retrospective study. 50 psychological interventions required for OIT related problems were selected consecutively in a Referral Centre in North‐Eastern Italy. All patients had a medical diagnosis of FA and were undergoing OIT or had just discontinued it. Data were collected from hospital records. A descriptive statistical analysis was performed.

**Results:**

66% of patients asked for psychological support for the initial phase (e.g., oral food challenge, first maintenance doses), 20% during the up‐dosing phase, 8% during maintenance and 6% after discontinuation. 70% of treatments were required mainly because of emotional problems including dysfunctional anxiety and mood disorders, increased distress and excessive worry and/or fear related to OIT; 20% because of difficulties in managing OIT; 10% because of eating difficulties; 50% of patients reported recent anaphylaxis. All patients reported improvement and felt the psychological intervention was helpful.

**Conclusion:**

It is recommended to evaluate the psychological needs in profiling patients and families suitable to OIT and offer specific psychological support when needed.

## 1 INTRODUCTION

Oral immunotherapy (OIT) is a promising treatment for food allergy (FA).^1^ Studies in patients and parents have demonstrated that completion of OIT, or reaching its maintenance phase, in general significantly improves both child and parents quality of life (QoL).^2^, ^3^, ^4^ In particular, the food anxiety and social and dietary limitation scores tend to decrease, while the emotional impact score did not.^2^, ^3^ Rigbi et al. found that, during OIT, patients' QoL improves in some individuals but deteriorates in others. Patients with impaired QoL at baseline improve significantly despite the treatment burden. Some patients with better QoL at baseline might deteriorate during OIT.^3^ OIT is a challenging process for patients and families. It is prolonged, ranging from several months to several years, and requires daily consumption of food the patients were taught to avoid and fear. Also, patients commonly have oral aversion to the allergenic food making it more difficult to consume on a regular basis.^3^ Moreover, adverse reactions are often experienced during treatment. Those range from mild reactions to anaphylaxis.^1^, ^5^ It is understandable that OIT can be a source of anxiety for patients and their families^6^: in particular the induction phase – during which patients are exposed to increasing amounts of food allergen until an allergic reaction develops, to establish the maximal tolerated dose has the potential to create anxiety in OIT participants.^7^ Anxiety, in turn, may affect patients' motivation and their ability to cope with the challenges of this extended treatment. Health care providers should be aware of the psychological challenges these patients and families face and know when to encourage them to seek psychological support, however there is no literature data on this specific issue.

This study aimed to investigate psychological needs and support offered to patients and their families undergoing immunotherapy assessing patients' main characteristics, reasons for seeking psychological support, OIT phase and related psychological difficulties, type and timing of treatments and patients' perception of the effectiveness of the intervention.

## 2 METHODS

This is an observational, retrospective study. Fifty psychological interventions required for OIT related problems were selected consecutively at the FA Referral Centre (FARC) of Padua University Hospital of Veneto Region (a Public Service in North‐Eastern Italy – about 5 million inhabitants) from October 2013 to October 2019. All treatments were held by a clinical psychologist with expertise in FA. The Centre offers a psychological health care service exclusively dedicated to patients with FA and their families to support the psychosocial burden of FA. All patient enrolled for the OIT were informed that specific psychological support was available. Patients involved in the study had a medical diagnosis of IgE‐mediated FA – on the basis of a clinical history with positive skin prick test (≥3 mm), in vitro specific IgE (>0.35 kU/L) and a positive oral food challenge (OFC) – and were either undergoing OIT or had just discontinued it. Data were collected from hospital records. A descriptive statistical analysis was performed to assess patients' characteristics, OIT phase and related psychological difficulties, type and timing of interventions and patients' perception of effectiveness.

The reasons why patients asked for psychological support were analysed and assigned to three categories: emotional problems (we used this ‘umbrella’ term to describe a diverse array of clinically significant symptoms and syndromes in which some kind of emotional alteration is involved such as dysfunctional anxiety and mood disorders, increased distress and excessive worry and/or fear),^8^ compliance, eating difficulties. Patients' perception of the effectiveness of treatments was measured through an adaptation of the additional follow‐up questions of the Strengths and Difficulties Questionnaire of Goodman for use after an intervention (available at http://www.sdqinfo.com/) as done in a previous publication.^9^ The study was performed in accordance with the European regulation regarding potential sensitive data and has been approved by the Padua University Hospital Ethics Committee. Authors intentionally avoided reporting detailed information about patients to guarantee anonymity and confidentiality.

## 3 RESULTS

From October 2013 to October 2019, 303 patients approached OIT. Of these, 55 (18%) were referred to the psychologist for difficulties related to the therapy. Among the latter, 50 (91%) undertook psychological treatment and constituted the sample of this study. They represent 16.5% of all patients who have undergone OIT in the considered period. In the sample 66% sought psychological care spontaneously, while 34% did it following the allergist's suggestion.

All participants came from North Italy and were White. They had a mean age of 18.02 (Standard Deviation 12.98); 66% were females, 8% came from a low‐income family, 6% had previous psychiatric comorbidity, 70% had multiple FA, 50% reported a recent anaphylaxis, all had adrenaline auto‐injector prescription.

**FIGURE 1:**
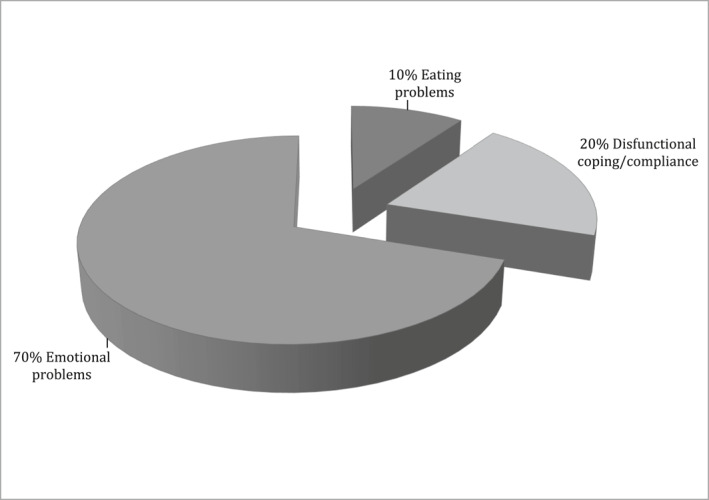
Why patients/families asked for psychological support in oral immunotherapy

**FIGURE 2:**
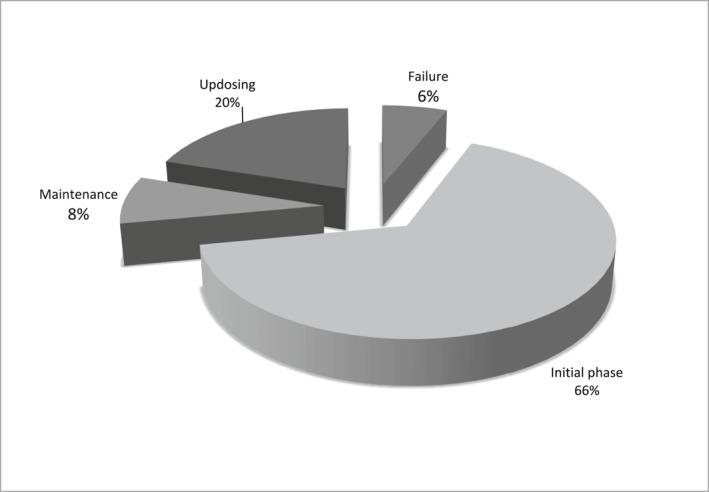
Oral immunotherapy phase in which patients/families ask for psychological support

With regard to OIT food, 36% was egg and 64% milk (Table 1). Overall, out of a total of 50 interventions, 36% were addressed to the whole family (patient undergoing OIT and parents), 26% to teenagers, 16% to patients' mothers, 14% to children and 8% to adults, as showed in Table 2. Concerning to the OIT phase, 66% of patients asked for psychological support for the initial phase (e.g., OFC, first doses of maintenance at home), 20% during the up‐dosing phase, 8% during maintenance and 6% after discontinuation due to reactions (Figure 1). With regards to the reason why patients asked for psychological support (Figure 2), 70% of treatments were required mainly because of emotional problems including dysfunctional anxiety, distress and mood disorders, excessive worry and/or fear related to OIT. Moreover 20% of treatments were held because of difficulties in managing OIT that included inadequate compliance and/or maladaptive coping strategies. 10% of the requests were expressed because of eating difficulties (excessive diffidence or disgust toward food) that interfered with the assumption of OIT foods. In two cases, after the psychological consultation it was agreed with the patient/parents and the medical staff not to start the OIT. Seven patients who were hesitant about starting OIT decided to start the treatment after psychological consultation. Five patients, who thought to drop out, decided to continue after psychological intervention.

**TABLE 1:** Frequencies of psychological interventions by OIT food and type of problem

OIT food	% of psychological interventions *N* = 50	Problems for which psychological intervention was required
Milk	64%	67% emotional
		5% eating
		28% compliance/coping
Egg	36%	72% emotional
		12% eating
		16% compliance/coping

Abbreviation: OIT, Oral immunotherapy.

**TABLE 2:** Frequencies of psychological interventions by recipient and type of problem

Recipients of the psychological intervention	% of psychological interventions *N* = 50	Problems for which psychological intervention was required
Families (OIT patient and parents)	36%	78% emotional
Patients' age = mean 11.61; standard deviation 4.47		11% eating
		11% compliance/coping
Patient's mothers	16%	75% emotional
Age = M 44.5; SD 7.17		25% eating
		0 compliance/coping
Children (5–12 years old)	14%	85% emotional
Age = M 8.77; SD 2.38		1% eating
		14% compliance/coping
Teenagers	26%	46% emotional
Age = M 14.5; SD 1.66		7% eating
		46% compliance/coping
Adult patients	8%	75% emotional
Age = M 24; SD 4.24		0 eating
		25% compliance/coping

Abbreviation: OIT, Oral immunotherapy.

With regard to type and timing of treatments, 52% of treatments consisted of psychological support (talk therapy, brief cognitive behavioural therapy [CBT], relaxation treatment – for example Jacobson Progressive Muscle Relaxation)^10^ – and Guided Affective Imagery (GAI),^11^ 38% counselling and psychoeducation, 10% psychotherapy (CBT); 82% of treatments were brief therapy (up to 8 sessions) and 12% mid/long‐term (9 to 32 sessions). Each session lasted 45 min. The type of treatment was decided according to general guidelines,^12^ FA literature data^13^ and clinical experience.

Data on patients' perception of the effectiveness of the intervention (Table 3) showed that all patients reported improvement (60% a bit better; 40% much better) and felt the intervention was helpful (50% quite a lot; 50% a great deal).

**TABLE 3:** Assessment of patients' perception of the psychological intervention effectiveness (*N* = 50)

How are your problems after the intervention?	Much worse	A bit worse	About the same	A bit better	Much better
	0	0	0	60%	40%
Has the intervention helped in other ways, e.g. providing information or making the problems more bearable?		Not at all	Only a little	Quite a lot	A great deal
		0	0	50%	50%

## 4 DISCUSSION

This is the first study, to our knowledge, aimed to investigate psychological needs and support among patients and their families undergoing food immunotherapy, assessing patients' main characteristics, reasons for seeking psychological support, OIT phase and related psychological difficulties, type and timing of treatments and patients' perception of the effectiveness of the intervention.

More than half of psychological interventions in our sample were requested at the initial phase of OIT. During the induction phase, patients undergo an initial OFC and are repeatedly exposed to increasing amounts of food allergen until the maximal tolerated dose of allergen is established. In this phase patients actually get a detailed picture of the facets and dangers related to the severity of their FA and, therefore there is higher potential of increased fear and anxiety.^7^ QoL in patients initiating OIT is significantly affected, not only by the severity of previous reactions, but also by their tolerated starting doses. This may reflect appropriate anxiety in the face of a more severe FA but might also affect patient motivation and coping ability during treatment.^7^ Psychological support can reduce excessive anxiety and distress and enhance the patient's personal and family resources.

Almost a quarter of treatments were required during the build‐up phase and a small part during maintenance. Oral immunotherapy is a lengthy and demanding therapy. It has been found that patients whose QoL was less affected by their FA at baseline subsequently experience a deterioration in QoL during OIT, probably due to the burden related to the treatment. These patients and families may need to be supported in motivation and compliance. It should be noted that a number of patients ask for psychological support after OIT discontinuation due to reactions. Oral immunotherapy can represent a great hope for patients and parents, therefore discontinuation can lead to dejection and decline in mood that may need psychological support.

Most treatments were addressed to the whole family. Oral immunotherapy requires preparing food in the right quantity, taking it regularly, respecting certain safety rules and observing and managing possible reactions. Especially for paediatric patients, these challenges involve and burden on the whole family daily and in addition to FA. The family can be considered as an entity that has to accommodate the requirements imposed by FA, and in this case also by OIT. Considering the family as a collective entity permits to support families in responding to conflicting circumstances, in order to create and sustain the daily routines in which they pursue their own goals and values, decreasing distress and improving the compliance.^14^ A considerable number of treatments were addressed to teenagers, especially for compliance or emotional problems. Oral immunotherapy requires motivation, self‐care behaviours and strict adherence. These aspects are particularly troublesome amongst adolescents. Jones et al. found that only 16% of teenagers were fully adherent to self‐management aspects of FA.^15^ Several developmental factors may contribute to the low adherence among adolescents with chronic conditions. For example, parent‐child conflict tends to increase during adolescence, as adolescents' desires for greater autonomy run counter to the need for parental supervision.^16^, ^17^ The demands of the adolescents' illness and therapy require them to comply with both parental and medical instructions. This doubling of authority, at a time where independence is typically sought, can lead adolescents to wilfully defy established rules^16^ with possible risk of dropout for OIT. Adolescents were found to be more receptive to a collaborative, empathic, patient autonomy granting and motivational approach.^16^ On the other side, adolescents who reported greater responsibility in their FA self‐management, also described feeling more anxious.^18^ Excessively anxious teenagers need to be supported especially in the initial phase of OIT. A number of treatments were addressed to mothers: this is in line with previous studies reporting that mothers are more involved in FA management and show higher scores than fathers for anxiety and distress.^19^, ^20^, ^21^ Finally, some interventions were addressed to children and to adults mainly for emotional problems at the initial phase. Based on findings, 70% of patients asked psychological care mostly for emotional problems that included dysfunctional anxiety, mood disorders, increased distress and excessive worry and/or fear related to OIT. These emotional difficulties can interfere with the compliance but also with the progress of the therapy. Anxiety expressions can mimic the symptoms of an allergic reaction (e.g., difficulty in breathing, abdominal pain) while a distress status can exacerbate some allergic manifestations (e.g., dermatitis and asthma).^6^, ^22^ Some patients can benefit from a psychological training aimed to alleviate dysfunctional anxiety and distress. Moreover 20% of treatments were held because of difficulties in managing OIT that included inadequate compliance and/or coping strategies. Inappropriate overconfidence and minimisation of the risk or, on the other side, high levels of anxiety and vigilance are associated with maladaptive strategies.^6^, ^17^, ^23^, ^24^, ^25^ Patients with an avoidant approach tend to forget to take their daily dose or not follow their doctor's directions for taking it. Instead those with an anxious approach tend to be excessively focused on the therapy and/or on possible reactions, showing great concern and distress that is counterproductive to continue the treatment.

Finally, 10% of treatments were requested because of eating difficulties such as excessive diffidence and fear or even disgust toward food that interfered with the assumption of OIT foods. Oral immunotherapy requires patients to eat the food that they have long been taught to carefully avoid or which in the past has been the source of even severe reactions. Furthermore, children with FA have shown low interest in tasting new foods^26^ and a common oral aversion to the allergenic food.^3^ In these cases, in addition to emotional support and CBT, the use of relaxation and GAI was found to be useful. These techniques aimed to decrease the levels of hyperactivation and distress, to draw out emotional fantasies and to facilitate the cathartic release of emotions that are present but painful or disturbing for the patient, leading to desirable changes in both affect and attitudes toward life situations.^11^

Moreover, 50% of patients reported a recent anaphylaxis. Anaphylaxis constitutes the most frightening allergic reaction, placing patients at high risk of death. This can motivate patients to start OIT, often with the aim of achieving a buffer against an unintentional reaction.^27^, ^28^ However, a recent study proved that a meaningful proportion of children and parents showed acute distress symptoms after food‐induced anaphylaxis^29^: this can interfere with the OIT pathway and, on the other side, the burden of therapy can worsen the distress condition. The physical, cognitive, and behavioural aspects of distress and anxiety that may be associated with anaphylactic risk must be addressed to ensure optimal psychological as well as medical outcomes.^6^, ^29^

With regard to type and timing of treatments, the majority of patients needed psychological support including talk therapy, brief CBT, relaxation treatment and GAI, in order to normalise and sustain a functional level of anxiety and to facilitate more adaptive approaches (emotional, behavioural, cognitive and social) and coping strategies, to alleviate feelings of distress and support patients and families in managing the possible challenges of OIT. A number of treatments consisted in counselling and psychoeducational intervention mainly focused on improving patients' awareness of their difficulties and strengths in dealing with OIT, promoting motivation and compliance. Only a small number of patients required psychotherapy (weekly sessions of CBT, 17 to 32 sessions) for fully diagnosed mental health disorders or subthreshold conditions. It is worth to note that all patients reported at the end of their treatment a moderate or great improvement of their situation. Half of the participants evaluated the intervention as very helpful. The majority of treatments consisted of brief therapy (up to 8 sessions) showing that a relatively little effort to support mental health can lead to a noteworthy improvement in the treatment pathway.

This exploratory analysis presents a number of shortcomings that have to be acknowledged. The investigation is very descriptive in its nature, although it first offers an overview of psychological needs, support and perception of its effectiveness among patients and families undergoing immunotherapy comparing clinical data from a tertiary level referral centre to literature. Effectiveness of the intervention was measured only through a brief patients' self‐report; further studies should use more specific and comprehensive instruments, as well as pre‐post and long‐term evaluations to quantify the improvement on patients' mental health.

Patients and families report perceived psychosocial benefits following OIT; however, there is also burden associated with the treatment itself.^30^ A small but substantial proportion of patients and parents experience significant psychological distress and/or maladaptive coping responses related to OIT which can interfere with the patients' and families' functioning but also with the OIT programs. Patients and families may have a variety of unique psychological needs related to dealing with OIT. A certain subset may require guidance or psychological support to reach a balanced approach, compliance and adherence to the therapy. This paper could help clinicians to recognise psychological needs and benefits of psychological support in food OIT in order to maximise the efforts and effectiveness of this treatment. It is recommended to consider the psychological needs in profiling patients and families suitable to OIT and offer specific psychological support when needed.

## CONFLICT OF INTEREST

Nothing to declare.

## ETHICS APPROVAL AND CONSENT TO PARTICIPATE

The study was performed in accordance with the European regulation regarding potential sensitive data and has been approved by the Padua University Hospital Ethics Committee. Parents and patients of age signed a written informed consent.

## AUTHOR CONTRIBUTIONS

**Laura Polloni:** Conceptualization; Data curation; Formal analysis; Investigation; Methodology; Project administration; Supervision; Writing – original draft; Writing – review & editing. **Antonella Muraro:** Conceptualization; Data curation; Formal analysis; Investigation; Methodology; Project administration; Resources; Supervision; Writing – original draft; Writing – review & editing. **Roberta Bonaguro:** Data curation; Writing – review & editing. **Alice Toniolo:** Data curation; Writing – review & editing. **Anna Ballin:** Data curation; Writing – review & editing. **Alberto Guarnaccia:** Data curation; Writing – review & editing. **Francesca Lazzarotto:** Data curation; Resources; Writing – review & editing.

## CONSENT FOR PUBLICATION

Parents and patients of age signed a written informed consent for their data to be used for scientific purposes including publications.

## Data Availability

The dataset used during the current study is available from the corresponding author on reasonable request.
